# Mapping the Exposome of Mental Health Outcomes to Enhance Population Salutogenesis

**DOI:** 10.31083/AP44314

**Published:** 2025-06-27

**Authors:** Melike Karaçam Doğan, Sinan Guloksuz

**Affiliations:** ^1^Department of Psychiatry, Karadeniz Eregli State Hospital, 67300 Zonguldak, Turkey; ^2^Department of Psychiatry and Neuropsychology, School for Mental Health and Neuroscience, Maastricht University Medical Center, P.O. Box 616 6200 MD Maastricht, The Netherlands; ^3^Department of Psychiatry, Yale School of Medicine, New Haven, CT 06511, USA

*“It is a mistake to think you can solve any major problems just with potatoes.”* [1]

Being among the most prevalent and chronic conditions worldwide and affecting millions of individuals globally, mental disorders have a complex etiology that involves a multitude of genetic and environmental factors. The level of complexity requires a holistic approach, and we proposed using the exposome paradigm in psychiatric research to extend the biopsychosocial framework beyond internal and social factors to include a broad spectrum of exposures, offering a deeper understanding of the “missing heritability” in psychiatric disorders [2].

The exposome covers all environmental exposures experienced by an individual from conception onwards to complement the genome in understanding complex diseases [3, 4]. The comprehensive framework for capturing a wide array of environmental exposures also aligns closely with transdiagnostic psychiatry. A specific environmental exposure, such as childhood trauma, can be recognized as a significant risk factor for multiple psychiatric conditions. Similarly, the cumulative environmental risk score for schizophrenia—exposome score for schizophrenia—is not only linked to psychosis but also correlates with broader psychopathology, functioning, and physical health outcomes [5, 6, 7]. In this regard, studies focusing on one environmental exposure and its effect on a single outcome fail to capture the complexity and interconnected nature of environmental factors [8, 9]. The transdiagnostic significance is further emphasized through the exposome-wide association studies (ExWAS), which enable hypothesis-free analyses to identify relationships between environmental exposures and a range of psychiatric outcomes. The hypothesis-free nature of ExWAS increases the chances of identifying novel environmental risk factors. ExWAS also has increased statistical power by applying advanced techniques that ensure more reliable and consistent identification of environmental factors that influence health outcomes [10]. The ExWAS approach has been applied to explore a range of specific mental health outcomes. We recently examined 294 exposures and in a comprehensive ExWAS of using data from UK Biobank [11]. This analysis revealed that exposures, particularly of those previously well-studied, such as childhood adversities, traumatic experiences, and cannabis use, were associated with multiple mental outcomes, including psychotic, bipolar manic, depressive and anxiety disorders, thereby further emphasizing the transdiagnostic relevance of these exposures in mental health [11]. We also demonstrated that unique relations between particular exposures and specific mental health outcomes might be present, such as time spent on computers being associated with neurodevelopmental disorders and childhood adoption linked to self-harm. An ExWAS examining 139 neighborhood-level environmental exposures has demonstrated that socioeconomic factors and safety are the most significant predictors of well-being [12]. Furthermore, these ExWAS can be combined with Mendelian Randomization methods to infer causality. In such a study, we demonstrated sexual assault victimization was a potentially causal risk factor for psychotic experiences, whereas cannabis use, worrying too long after embarrassing situations, and physical assault appeared to be aftereffects of psychotic experiences [13].

By mapping the exposome, we can identify potentially modifiable environmental exposures to inform public health policies and population-level interventions. ExWAS can further help in understanding potential protective factors that may promote resilience. Altogether, this aligns perfectly with the population salutogenesis framework, which emphasizes the importance of improving general health and well-being rather than solely focusing on disease treatment [14]. This salutogenesis approach prioritizes the promotion of healthy behaviors, the elimination or reduction of risk factors, while increasing protective factors, such as social support, physical activity, and access to green spaces, which contributes to the proactive building of resilience, ideally before the need for early intervention by specialized professionals even arises [14].

Integrating salutogenesis into public mental health initiatives presents an important opportunity to improve mental well-being at the population level. This approach encourages the understanding that mental well-being is more than just the absence of a diagnosable mental illness; it is about enhancing the quality of life for all individuals, not just those diagnosed with psychiatric conditions. School-based programs that focus on building resilience, such as teaching coping strategies and social skills, workplace well-being initiatives that prioritize stress reduction, and community-wide efforts to reduce the mental health stigma can be essential components of these public health initiatives. It is equally important to implement policies that guarantee everyone has fair access to mental health services. This approach will also support individuals with existing mental illnesses in improving their health; in fact, those with psychiatric disorders are demonstrated to be more likely to take action following a health promotion campaign [15]. Salutogenic principles should not only be applied at the individual level but also on a societal scale, addressing broader issues such as socioeconomic inequality and public safety through regulatory interventions [14].

A transformative impact on population mental health is not an unattainable goal; it only requires a comprehensive approach that involves dissecting the modifiable factors within the complex, interconnected exposome and strategically addressing them through public health interventions and policy reforms.
